# *Chlamydia Trachomatis* Infection: Their potential implication in the Etiology of Cervical Cancer

**DOI:** 10.7150/jca.58582

**Published:** 2021-06-11

**Authors:** Xingju Yang, Anam Siddique, Abdul Arif Khan, Qian Wang, Abdul Malik, Arif Tasleem Jan, Hassan Ahmed Rudayni, Anis Ahmad Chaudhary, Shahanavaj Khan

**Affiliations:** 1 Department of Nursing, Jinan People's Hospital Affiliated to Shandong First Medical University, Jinan, Shandong 271199, China.; 2 Department of Biosciences, Shri Ram Group of College (SRGC), Muzaffarnagar 251001, India.; 3 Division of Microbiology, Indian Council of Medical Research-National AIDS Research Institute, Pune, Maharashtra, India.; 4 Department of Obstetrics and Gynecology, Jinan Fifth People's Hospital, Jinan, Shandong, 250022, China.; 5 Department of Pharmaceutics, College of Pharmacy, P.O. Box 2457, King Saud University, Riyadh 11451, Saudi Arabia.; 6 School of Biosciences and Biotechnology, Baba Ghulam Shah Badshah University, Rajouri 185236, India.; 7 Department of Biology, College of Science, Imam Mohammad Ibn Saud Islamic University (IMSIU), Riyadh 11623, Saudi Arabia.; 8 Department of Health Sciences, Novel Global Community Educational Foundation, Australia.

**Keywords:** bacteria, *C. trachomatis*, infection, *C. trachomatis* proteins, cervical cancer, etiology

## Abstract

Pathogenic bacterial strains can alter the normal function of cells and induce different levels of inflammatory responses that are connected to the development of different diseases, such as tuberculosis, diarrhea, cancer etc. *Chlamydia trachomatis* (*C. trachomatis*) is an intracellular obligate gram-negative bacterium which has been connected with the cervical cancer etiology. Nevertheless, establishment of causality and the underlying mechanisms of carcinogenesis of cervical cancer associated with *C. trachomatis* remain unclear. Studies reveal the existence of *C. trachomatis* in cervical cancer patients. The DNA repair pathways including mismatch repair, nucleotide excision, and base excision are vital in the abatement of accumulated mutations that can direct to the process of carcinogenesis. *C. trachomatis* recruits DDR proteins away from sites of DNA damage and, in this way, impedes the DDR*.* Therefore, by disturbing host cell-cycle control, chromatin and DDR repair, *C. trachomatis* makes a situation favorable for malignant transformation. Inflammation originated due to infection directs over production of reactive oxygen species (ROS) and consequent oxidative DNA damage. This review may aid our current understanding of the etiology of cervical cancer in *C. trachomatis*-infected patients.

## Introduction

Cancer is very complex and deadly disease worldwide. In 2019, 1,762,450 fresh cancer cases and 606,880 cancer fatalities were recorded in the United States according to National Center for Health Statistics 1. Every year, more than half a million females are detected with cervical cancer which causes about 300000 deaths globally 2. Comparative results showed that the death rates were 2-fold higher in the poorest counties in case of cervical cancer during 2012-2016 1. Various factors are associated with growth and development of various types of cancer including mutagenic chemical, exposure of ionized radiation, diets, tobacco, infections, etc. 3. Infections of various microbes are considered as an important etiological factor of progression and development of various types of cancer. Different viruses such as human T‑lymphotropic virus 1, Epstein‑Barr virus (EBV), Kaposi's sarcoma associated herpes virus, hepatitis B virus (HBV), hepatitis C virus (HCV), human papilloma virus (HPV) and pathogenic strains of bacteria including *E. coli*, *Chlamydia pneumoniae, Mycoplasma hominis*, *Helicobacter pylori*, are associated with the growth and development of various types of cancer 4-11*.* The infections of various microorganisms stimulate the innate or adaptive immune response which may cause chronic inflammation leading to induction of pro‑tumorigenic effect.

About 20% of malignancies are associated with different microbial infections 12. Many evidences have confirmed that different pathogenic bacteria are linked with the growth of various types of cancer 7, 13. Gram-negative obligate intracellular *C. trachomatis* bacteria are connected with genital tract and ocular infections in humans. Cervical cancer is the most common type of gynecological cancer in women. Various evidences have shown that pathogenic bacterial strains induce different levels of inflammatory responses which have associated with growth of various types of diseases such as tuberculosis, diarrhea, cancer etc. Etiology of cervical cancer is highly complicated and multifaceted. Though, the particular cause of cervical cancer remains unclear, nevertheless, various possible factors have been identified. The connection of infectious microorganisms in the growth and development of different types of cancer have focused the attention of scientists in few past years. *C. trachomatis* is an intracellular obligate gram-negative bacterium which has been connected with the growth of cervical cancer. In women, the infection of sexually transmitted C. trachomatis bacteria is very common. Whereas some epidemiological studies show a high rate of *C. trachomatis* infection in women with cervical cancer, others do not 14, 15. For instance, Tungsrithong et al. (2014) found no effect, or only a marginal effect, of *C. trachomatis* infection on the rate of cervical cancer in case control study from Thailand 16. Hence, the issue of whether the infection of C. trachomatis enhances the chance of growth of cervical cancer has so far not been cleared and is still a subject of research 16. The establishment of causality and the underlying mechanisms of carcinogenesis of cervical cancer associated with *C. trachomatis* remain unclear. Although, it has observed that bacterial-host interactions are very diverse, their accurate associations for the growth and development of cancer are not well cleared. Particularly, various pathogenic strains of bacteria have been demonstrated to alter the normal functions of the host cell through different strategies during the course of infection. However, it is not understood, how *C. trachomatis* plays a crucial role in the growth of cervical cancer. In the current review, we will discuss how the infection of *C. trachomatis* involved in carcinogenesis of cervical cancer and how the *C. trachomatis* effector proteins and protein toxins enigmatically interact with host cells. Moreover, we will try to discuss how such encounters can result for the growth of cervical cancer through alteration in normal host cell signaling pathways. We therefore aimed at enlightening the latest updates about the relationship between *C. trachomatis* and cervical cancer. We summarized and explained the underlying molecular mechanisms of chronic inflammation and their implications in the growth and development to cervical cancer.

## Role of infections in the etiology of cervical cancer

Infections of various microorganism including viruses, parasites and bacteria, have been categorized as possible risk factors for the progression and development of different type of cancers. The infectious agents are associated with the growth and development of about a fifth of different cancers in humans worldwide. An update of their respective involvement to the worldwide burden of cancer is assured. Breast cancer and cervical cancer are the most common neoplastic diseases which mainly affect the females and leads to high worldwide mortality. It is suggested that the *C. trachomatis* act as a cofactor with the HPV for influencing development of cervical cancer. Studies encompassing the last two decades have shown overwhelming evidences that infection of sexually-transmitted human papillomavirus (HPV) is connected with the growth cervical cancer 10, 17. Nevertheless various studies have demonstrated that C trachomatis infection is also linked with the growth and development of cervical cancer 18.

Seven diverse viruses have been connected to the growth of different types of human cancer, such as human papillomavirus, Epstein-Barr virus, human T-cell lymphotropic virus, hepatitis B virus, hepatitis C virus, Merkel cell polyomavirus and Kaposi's sarcoma herpes virus. Although infection of human papilloma virus (HPV) is considered one of the most essential factor for the growth of cervical cancer 19 but various other factors also connected for the development of cervical cancer which include infection of *C. trachomatis*, early age at first coitus, premarital sexual and extramarital sexual activity, early age pregnancy, multiple sexual partners of the male and females, husbands, and uncircumcised sexual partners 20, 21. Though, the infection of HPV is generally transient but little percentage of females showed persistent infection which has connected with the development of cervical cancer. Different strains of HPV play important role in the progression and development of cervical cancer. However, researchers have discovered >100 types of different HPV but about 15 genotypes of HPV have shown the potential for the growth of cancer. Every HPV genotype proceeds as an independent infection, with different level of carcinogenesis. HPV genotype 16 and genotype 18 is found with pre-cancerous cervical lesions and approximately 70% of cervical cancer 22, 23. Most of the persons are infected with the infection of HPV soon after the beginning of sexual activity as the virus has the potential of transmission by sexual contact.

These causes include physical stress, immune response and other sexually transmitted bacterial infections. The bacterial infection may act as a powerful co-factor for the transformation in host cell for the progression of cervical cancer. The infection of sexually transmitted bacterium *C. trachomatis* is generally not symptomatic, nevertheless develops diverse syndromes including endometritis, infertility, pelvic inflammatory disease, and cervicitis. It is demonstrated that *C. trachomatis* helps in the pathways related to cell proliferation and inhibits the process of program cell death. Moreover, *C. trachomatis* could impede the immunological response through reduction of antigen presenting cells, decreased the cell-mediated immunity and stimulate the chronic inflammation which may allow the HPV persistence. In general, the growth and development of cancer is a complex multistep procedure, which is also factual for bacterial mediated progression of cancer. The specific pathogenic strains of different types of bacteria exert enigmatic effects on various pathways of host cell 6, 7, 15, 24. Bacteria may alter the normal functions of host cells using different strategies including mutations, altered regulations of various biochemical pathways, cell proliferation, apoptosis which are connected with the initiation and growth of cancer.

### Bacterial infection and cervical cancer

In the last decade, many reports have shown the role of *C. trachomatis* bacterium in the growth and development of cervical cancer 15, 25, 26. *C. trachomatis* related cancer in females demonstrate eight different mutational signatures (http://cancer.sanger.ac.uk/cosmic/signatures). One of the most prominent attribute is faulty homologous recombination (HR) repair. Although, the mechanism of these mutational processes is not well understood, and therefore it is fascinating to know that the infection of obligate intracellular bacterium *C. trachomatis* directs DNA double-strand breaks (DSBs), alters the various cellular functions in host cells and changes the normal functioning of signal transduction pathways which is involved in immune system activation, preserving of genomic integrity and induction of apoptosis 27-29. A study showed that infection of *C. trachomatis* down-regulate the expression of p53 tumor suppressor gene in infected cells 30.

DSBs demonstrate the most dangerous form of DNA damage due to their unrepaired nature in general and therefore it may cause the risk of instability in the genome of organism 31. DNA damage response (DDR) delays the genomic instability and increase the frequency of mutations 31, 32. In our recent studies, we have illustrated the potential involvement of bacterial proteins in the growth and development of different types of cancer through alteration in normal functioning of different pathways 6, 7, 24. The host cells protected from cell death-inducing stimuli during the infection of *C. trachomatis* through adapting various strategies including extensive host DNA damage, dramatic depletion of p53, a tumor suppressor, which may cause a high risk of carcinogenesis 33, 34. A recent study discloses that plasmid-encoded protein Pgp3 of *C. trachomatis* inhibits the process of apoptosis through the activation of the PI3K/AKT signaling pathway in HeLa cells 35. Pgp3 has the potential to stimulate the infected cell for the formation of pro-inflammatory cytokines through Toll-like receptor 2 (TLR2) signaling pathway and activation of NALP3 inflammasome which suggest that the Pgp3 of *C. trachomatis* can alter the signaling pathways of infected host cells 36, 37. Various proteins of *C. trachomatis* may also alter the normal functioning of various pathways of infecting cervical cells during infection using different strategies.

### *Chlamydia* infection and risk of cancer

*Chlamydia* is another bacterial genus which is also proposed to be involved in cancer. However, contradictory evidences exist, but the implication of *C. trachomtis, C. psittaci, C. pneumoniae* is proposed in cervical cancer 25, ocular adnexal lymphoma 38, and in lung cancer 7, 39. Although exact role of *Chlamydia* in the carcinogenesis is still a subject of discussion, the occurrence of nucleomodulins in these organisms supports their role in the etiology of cervical cancer. *Chlamydia* has the ability to alter the chromatin structure due to the presences of SET domain proteins. The implication of SET domain proteins in the etiology of cancer has reported previously 40, which has showed that some SET domain proteins detected in *Chlamydia* with the capability to modify chromatin of host. Moreover, *Chlamydia* is identified to contain various other proteins with the potential to target the nucleus of host during infection. *C. trachomatis* has CT621 proteins, which is localized to cytoplasm and nucleus of host by type 3 secretion system (T3SS) 41. *C. psittaci* has SINC a T3SS protein, which is translocated to host cell inner nuclear membrane 42. There are several other proteins of Chlamydia with no known potential function are discovered to localize in host cell nucleus. Perhaps the upcoming advanced research will reveal the enigmatic role of *Chlamydia* in the growth and development of cancer with molecular study of nucleomodulins.

## *Chlamydia trachomatis* and cervical cancer

The 3^rd^ most common cervical cancer is considered as the 4^th^ leading source of cancer death globally in females. Although various factors are connected with the growth of cervical cancer, the infection of Chlamydia is also proposed for growth and development of cancer. Different proteins of C. *trachomatis* have the potential to promote the growth of the cervical cancer.

### Possible implications of *C. trachomatis* proteins in the etiology of cervical cancer

Bacteria have the potential to alter the different regulatory molecules of infected cell during infection for their survival. Various bacterial proteins can modify the normal pattern of gene expression and normal functions of different proteins and enzymes of infected cell through the disturbance in various protein-protein interactions, program cell death, cytoskeletal rearrangement etc. 43, 44. The obligate gram-negative intracellular pathogen *C. trachomatis* is infecting the epithelial cells of genital tract. Earlier studies have been showed that the infection of *C. trachomatis* enhanced the risk of cervical cancer through alteration in proteome of infected cells. 45-47. It has been observed that *C. trachomatis* proteins targeted into the cytoplasm of host which include deubiquitinating enzymes and several proteases. This advocates that the infection of *C. trachomatis* directly alters the infected host cell protein turnover nevertheless, the information of targeted proteins and their functions remain mostly not clear. Therefore, we may suggest that some proteins of *C. trachomatis* can target various sub-cellular compartments, such as nucleus, endoplasmic reticulum and mitochondria. Various sub-cellular targeted proteins can exert adverse effects which may inhibit various essential biological functions and responsible for the growth and development of cancer 48. It has observed that the infection of *Chlamydia* on cultured cells causes multinucleation which directs the chromosomal instability 49. Multinucleated cells are commonly found in most of the solid tumors which contribute in the condition of aneuploidy and chromosome instability 50. Recent report showed that *C. trachomatis* alters PP2A signaling to suppress Ataxia-telangiectasia mutated (ATM) activation which is responsible for high-fidelity repair of DNA double-strand breaks (DSBs) 26.

### Possible erroneous in DNA binding proteins and cervical cancer

**Figure 1:**
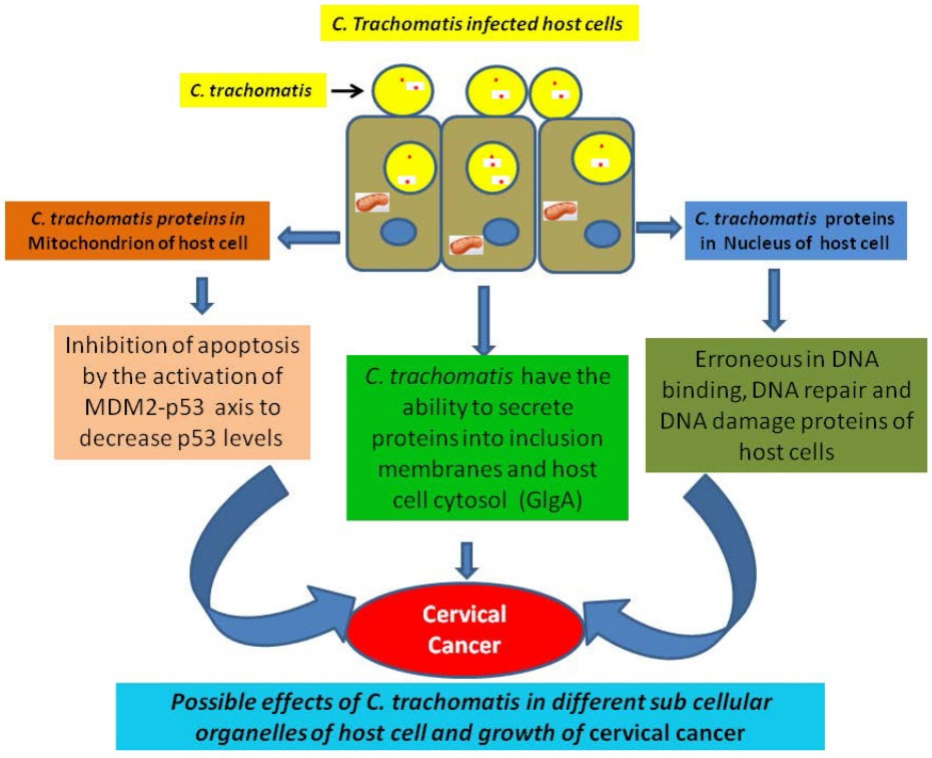
Alteration in normal functions of different pathways of *C. trachomatis* infected cell and growth of cervical cancer.

DNA binding proteins have crucial role in the growth and development of cancer. For instance, various DNA binding proteins such as CpG binding proteins triggers the growth of cancer by the process of methylation 51. Similarly chromodomain helicase DNA binding protein 5 is associated with tumour suppression, and mutation in this protein inhibits its function and enhance the growth of breast cancer 52. In a recent study the DNA binding damage-specific DNA-binding protein 2 (DDB2 protein) identified which is involved in development of cancer through facilitating the DNA nucleotide excision repair (GG-NER) in human cells 53. The existence of DNA binding proteins of human and *C. trachomatis* in the nucleus provides competitive opportunity for both proteins to bind with the target molecules. This competitive environment may affect the binding of normal human proteins and increase the risk of cervical cancer. The normal functioning of different pathways of *C. trachomatis* infected cell are altered which may direct the growth of cervical cancer (Fig. 1).

Implication of DNA binding inhibitor proteins in growth and progression of cancer has been confirmed in ovarian cancer, where over expression of inhibitor of DNA binding protein (ID-1) directs the growth of ovarian cancer 54. We have indicated several nuclear targeting protein (such as DNA gyrase subunit A, RNA polymerase factor sigma 54, DNA directed RNA polymerase subunit beta and mutL protein) of *C. trachomatis* which may be possibly connected with the growth of cervical cancer. These DNA binding proteins may be involved in the growth of cervical cancer and must be investigated further.

### Possible alteration in the DNA damage proteins and cervical cancer

*C. trachomatis* has the potential to stimulate the process of DNA damage through ROS mechanism. This mechanism directs double stand brakes and helps to generate senescence-mediated heterochromatin foci. Chumduri et al, (2013) showed that the infection of *C. trachomatis* suppress the double stand breaks repair function in infected cell. The cells infected with *C. trachomatis* divide more efficiently due to high level of oncogenic potential from CyclinE, ERK, and SAHF which direct to the growth of cancer 28. Moreover, many DNA damaging endonucleases are also observed which are associated to the growth of cancer, such as primary gastric cancer and lymph node metastasis is associated with the overexpression of LINE-1 endonuclease 55, 56. As illustrated in above paragraph, the host DNA damage occurrs in *C. trachomatis* infected host cell 28 and we can predict the nuclear targeting of various *C. trachomatis* proteins which have the capability to damage the DNA (Fig. 1). We have predicted of *C. trachomatis* MutS a DNA mismatch repair protein (Accession No. AAX51072) in the host cell nucleus by Hum-mPLoc Predictor. Nevertheless the prediction results required the validation through wet lab experiments and their implication the growth of cervical cancer.

### Possible erroneous DNA repair and cervical cancer

The important role of DNA damaged and/or repair proteins are to identify and repair the errors in single-base mismatch nucleotide which may arise during the process of DNA replication. The error recognition and following activation of the DNA damage repair mechanism depends on the complex of the enzymatic proteins which includes MuH MutS, and MutL 57. The MutS complex has the capability to identify mismatched nucleotides and attach to the damaged DNA. Alteration in DNA damaged and/or repair proteins are believed one of the potential etiological factors for the growth of cancer. The erroneous DNA repair directs either mutation or chromosomal aberrations which may directly leads to malignant transformation through affecting the tumor suppressor genes and/or oncogenes. Alteration in DNA mismatch repair proteins may direct microsatellite instability, a general event of progression and development of cancer 58, 59. For instance, mismatch DNA repair protein MutS is associated with the growth of colon cancer 24, 60. We have also predicted the nuclear targeting potential in *C. trachomatis* DNA mismatch repair protein MutL. As MutS is a DNA repair linked protein, the abnormality in DNA repair can direct growth of cancer. It has previously proposed that *C. trachomatis* suppresses DNA repair activity and induces DNA damage in host cells, but this suppressed DNA repair involved double stranded breaks 28. The upcoming advanced research on the role of nuclear targeted *C. trachomatis* proteins in suppression of mismatch DNA repair activity can certainly open new insights on the role of *C. trachomatis* in the development of cervical cancer (Fig. 1).

### Inhibition in apoptosis during infection of *C. trachomatis*

The infection of sexually transmitted* C. trachomatis* is considered the most important cause of infertility 61. Further, the infection of *C. trachomatis* is connected with the growth of invasive cervical cancer. Gonzalez et al., (2014) has showed that Chlamydia hampers the process of apoptosis in host cell through activating the MDM2-p53 axis to reduce p53 levels 62. Moreover the bacteria Chlamydia have the potential to exploiting the functional interdependence between cell survival and metabolic pathways 30, 63. Various types of cancers have demonstrated with this process, where activation of oncogenes or loss of tumor suppressors helps to direct the metabolic reprogramming through increased glycolysis and nutrient uptake.

### Delivery of *C. trachomatis* protein in cytoplasm of infected host cell

Similar to other gram-negative bacterial strains, *C. trachomatis* utilizes the type III secretion system (T3SS) to transfer different virulence effectors proteins into infecting host cells. These effectors proteins prompt bacterial uptake, survival and replication within the host cell. It has observed that glycogen synthase (GlgA) of C. trachomatis is secreted into cytoplasm of host cell. In fact, GlgA secretion into the inclusion lumen and cytoplasm of host cell has been confirmed 64. The synthesis of glycogen is stimulated in hypoxia stage through the hypoxia-inducible factor which helps in the survival of cancer cell.

## Inflammation in carcinogenesis of cervical cancer

Cancer micro-environments have the potential to alter the functions of the different immune cells as per requirement. In immune-elimination state, active cells of immune system eradicate transformed cells. However, during the establishment of cancer, the cells of innate and adaptive immunity provide protection to transformed cells and help for the fulfillment of their requirement. In between the state of elimination and establishment, equilibrium and escaping state control the response of immune cells. Epidemiological associations indicate that *C. trachomatis* serotype G is most strongly associated with development risk for cervical squamous cell carcinoma 18. It is showed that *C. trachomatis* acts as immuno-modulator, stimulates cervical inflammation, in lower genital tract decreases antigen presenting cells and have the ability to damage the mucosal barrier 10. Reports demonstrated that the existence of antibodies to *C. trachomatis* is connected with increased risk for squamus cell carcinoma 17, 65. Although various cohort studies showed that infection of HPV is connected with the growth of cervical cancer, but it is also observed that the infection of *C. trachomatis* solely connected with the growth of cancer 23, 66.

### Infection mediated immune response and cancer

The bacterial infection acts as a potential factor to alter the normal functioning of host cell through various strategies. The infected host cells activate different defensives pathways against to pathogenic infection. The intracellular infection of bacterium* C. trachomatis* is responsible for the activation of host immune system during infection. Indeed, multiplication of *C. trachomatis* is based on these changed proteins such as Pin 1 and Men 1, which involves the regulation of host transcription factor AP-1 for cell survival, stress and inflammation 45.

**Figure 2:**
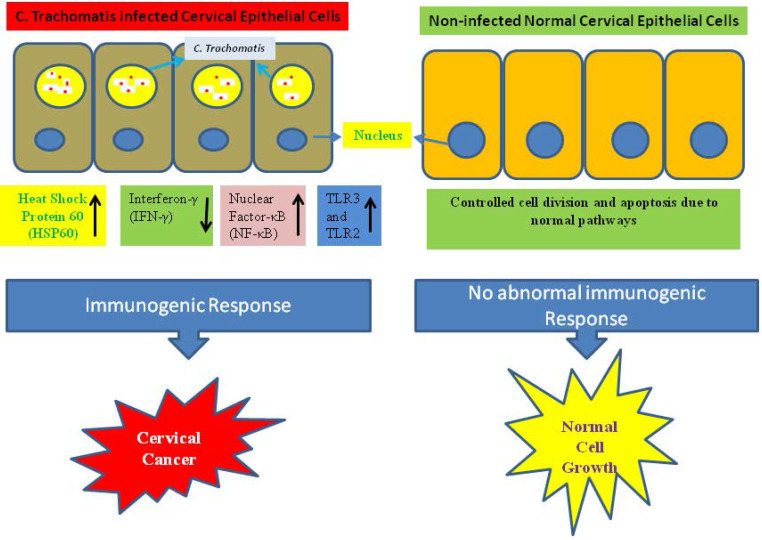
Abnormal immunogenic responses help in growth and development of cervical cancer during *C. trachomatis* infection.

A probable molecular mechanism in growth of cervical cancer is the infection-associated inflammatory response during the infection of *C. trachomatis* which helps to the production of reactive oxidative metabolite, enhances cytokines expression, chemokines, and growth and angiogenic factors. On the other hand during the infection of *C. trachomatis* reduced cell-mediated immunity, and the generation of free radicals. These alterations can cause damages to DNA and impair the function of DNA repair which may increase the genetic instability 67. The C. trachomatis infection interrupts N-cadherin-dependent cell-cell junctions and originated the breakdown of the complex of N-cadherin/β-catenin in primary cultures of HeLa cells and cervical epithelial cells 68. In a study, imbalance has observed in Matrix metalloproteinases-9/Reversion-inducing Cysteine-rich protein with Kazal motifs (RECK) during cervical inflammation stimulated by the infection of C. trachomatis which may play an important role in the carcinogenesis of cervical tissue 69. Additionally, the infection of C. trachomatis activated the chromosome segregation defects and synthesis of supernumerary centrosomes, triggered multipolar mitosis, dynamically supported chromosome instability, resulting multinucleation, and thereby facilitate the phenomenon of transformation and tumor growth and development 70, 71. Similarly it was revealed that infection of C. trachomatis in mice triggered considerable enhancement in cell proliferation within the cervical epithelium 72. Immunogenic responses act as a possible factor for the growth of cervical cancer (Fig. 2).

### Role of B and T cell in cervical cancer during infection of C. trachomatis

C. trachomatis is an intracellular obligate bacteria which is the common pathogen to cause sexually transmitted infection (STIs) and connected with the growth and development of cervical cancer 46. It is connected with ectopic condition of pregnancy, pelvic inflammation, and sterility in various cases, and is also involved to enhance the possibility of cervical neoplasia with slow growth cycle 73. The infection of C. trachomatis is targeted by T and B cells of innate immunity. Defensive immune responses manage the bacterial infection while pathological reactions direct to chronic inflammations 74.

### C. trachomatis infection and innate immunity

The process of carcinogenesis has triggered by different factors and events. Bacterial infection may promote the chronic inflammation, which acts as an important factor for the growth of cancer. Various case‑control reports have shown a connection between C. trachomatis infection and cervical cancer 15, 25, 73, 75. First line of host defense is provided by the mucosal epithelium of the genital tract. The innate immunity is triggered while the C. trachomatis entered into the mucosal epithelium through the detection of pathogen-associated molecular patterns (PAMPs) including Toll-like receptors (TLRs). Although C. trachomatis lipopolysaccharides can be identified through TLR4 but TLR2 is more important for the signaling of pro-inflammatory cytokine production 76. This condition directs the production of pro-inflammatory cytokines including tumour necrosis factor-alpha (TNF-α), interleukin-1 (IL-1), IL-6, and granulocyte-macrophage colony-stimulating factor (GM-CSF) 77. The role of such cytokines have been observed in the growth and development of cancer, invasion and metastasis 78. Moreover, chemokines including IL-8 can enhance recruitment of innate-immune response control cells including dendritic cells (DCs), natural killer (NK) cells, macrophages, and neutrophils that consecutively generate more proinflammatory cytokines to restrict the growth of C. trachomatis. Matrix metalloproteases (MMPs) is released by the infected mucosal epithelial cells which contribute in tissue proteolysis and remodeling. Neutrophils also release MMPs and elastases that contribute to tissue damage. Interferon gamma (IFN-γ) is produced by NK cells which pushes CD4 T cells toward the Th1-mediated immune response. The C. trachomatis infected cells are infiltrated by a mixture of CD8, CD4, B cells, and plasma cells 74, 79, 80. DCs play important role in processing and presenting of C. trachomatis antigens to T cells surface and thus connecting innate and adaptive immunity.

### C. trachomatis infection and adaptive immunity

The infection of C. trachomatis is tried to control by CD4 and CD8 cells 81. A study showed that depletion in CD4 cells, are not capable to eliminate the infection of C. trachomatis in B cell-deficient mice 82. Nevertheless, another report demonstrated that transfer of *Chlamydia* specific monoclonal antibodies into B-cell deficient and CD4 depleted cells re-established the capability of these mice to control a secondary infection of C. trachomatis 82. This phenomenon shows a powerful synergy between CD4 and B cells in the adaptive immune response to C. trachomatis. The C. trachomatis-specific antibodies are produced by B cells to fighting with the pathogens.

In contrast, the CD8 cells of immune system generate various interleukins such as IL-4, IL-5, and IL- 13 which do not emerge to defend against *Chlamydia* infection and may even indirectly increase the load of *Chlamydia* through inhibiting the protective response of CD4. This outcome proposes that a biased immune response toward Th1 occurrence defends against the chronic infection 74. The pathogenic response to C. trachomatis can result in inflammatory damage due to either weak or failed action of the Th1 resulting in an exaggerated Th1 response or chronic infection. On the other hand, chronic infection can arise if the response of Th2 dominates on the Th1 immune response and consequence activate the autoimmunity and cell damage which has triggered the tissue inflammation. This Inflammation enhances the heat shock protein (HSP) expression in host, which stimulate IL-10 production through auto-antibodies during the course of C. trachomatis infection 83. It has demonstrated that the cells of cervical epithelium express an efficient inflammasome which directs to activation of caspase-1 by a procedure involving the NOD-like receptor family member NLRP3 along with inflammasome adaptor protein during C. trachomatis infection. Therefore, it is required to expand the screening of *C. trachomatis* infection and punctually treated the *C. trachomatis* infected women, specifically those which have the infection of human papilloma virus. This strategy will help to prevent cervical cancer.

## Conclusions and future prospects

In conclusion, the current systematic review illustrate that the women infected with infection of *C. trachomatis* have an increased risk for the development of cervical cancer through different strategies. These include alteration in the normal functioning of different pathways such as apoptosis, DNA repair system, protein folding during the infection of *C. trachomatis* and their protein targeting in different sub cellular compartments of host cell. Different processes are involved in growth and development of malignancy during host pathogen interactions. The mechanism of *C. trachomatis* associated cervical cancer remains unclear and inadequate therapeutic approaches are available to treat and manage the cervical cancer. Host pathogen interaction is a very complex mechanism involving a range of molecules such as nucleomodulins. Nucleus is an important sub-cellular organelle of the cell and has key role in oncogenesis. Controlling of this cell organelle by pathogen is a crucial phenomenon and must be considered to understand the implications of bacteria in carcinogenesis. Though, various techniques are available to identify nuclear targeting of certain protein and its consequent effects on the functions of nucleus, but it is very difficult to evaluate complete bacterial proteome for their nucleomodulins potential. Several in-silico approaches are also accessible to balance this problem through deciphering the nuclear targeting of certain proteins rapidly. The present research indicates that certain specific proteins of C. trachomatis can be an important target in cervical cancer etiology and their knowledge will help us to plan suitable management strategies against cancer.
